# Designing Functional Bionanoconstructs for Effective *In Vivo* Targeting

**DOI:** 10.1021/acs.bioconjchem.1c00546

**Published:** 2022-02-15

**Authors:** Aisling Fleming, Lorenzo Cursi, James A. Behan, Yan Yan, Zengchun Xie, Laurent Adumeau, Kenneth A. Dawson

**Affiliations:** † Centre for BioNano Interactions, School of Chemistry, University College Dublin, Belfield, Dublin 4, Ireland; ‡ UCD Conway Institute of Biomolecular and Biomedical Research, School of Biomolecular and Biomedical Science, University College Dublin, Belfield, Dublin 4, Ireland

## Abstract

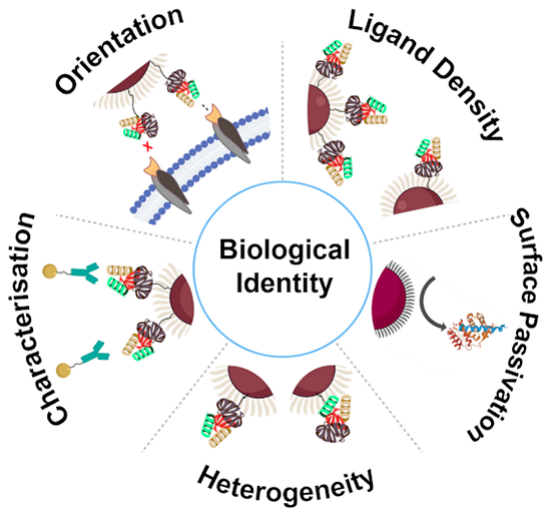

The progress achieved over the last three decades in the field of bioconjugation has enabled the preparation of sophisticated nanomaterial–biomolecule conjugates, referred to herein as bionanoconstructs, for a multitude of applications including biosensing, diagnostics, and therapeutics. However, the development of bionanoconstructs for the active targeting of cells and cellular compartments, both *in vitro* and *in vivo*, is challenged by the lack of understanding of the mechanisms governing nanoscale recognition. In this review, we highlight fundamental obstacles in designing a successful bionanoconstruct, considering findings in the field of bionanointeractions. We argue that the biological recognition of bionanoconstructs is modulated not only by their molecular composition but also by the collective architecture presented upon their surface, and we discuss fundamental aspects of this surface architecture that are central to successful recognition, such as the mode of biomolecule conjugation and nanomaterial passivation. We also emphasize the need for thorough characterization of engineered bionanoconstructs and highlight the significance of population heterogeneity, which too presents a significant challenge in the interpretation of *in vitro* and *in vivo* results. Consideration of such issues together will better define the arena in which bioconjugation, in the future, will deliver functional and clinically relevant bionanoconstructs.

## 1 Introduction

Over the last 30 years, bioconjugation has emerged as a cornerstone of medical research and biotechnology. Motivated by the desire to augment the properties of biomolecules, bioconjugation strategies are employed in a diverse range of applications including the study of biomolecules and their interactions, diagnostics, drug delivery, and bioimaging.^1−5^ The conjugation of biomolecules to nanomaterial surfaces to produce functional bionanoconstructs, in particular, has been pursued for a multitude of purposes, including analyte isolation and extraction^6^ and biosensing.^7−9^ A key underlying agenda on this front has been the desire to impart specific biological identities to nanomaterials, thereby advancing their role in biomedical applications.^10−20^

**Figure 1:**
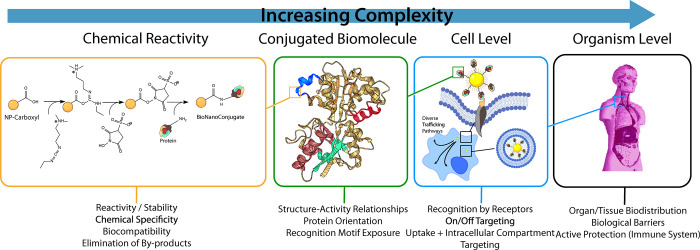
Levels of complexity in bionanoconstruct formation. The chemical reactivity of particular moieties found within biomolecules, as well as their exploitation in the selective formation of stable bionanoconstructs, represents the best-studied and most understood of these levels. At the level of the conjugated biomolecule, consideration must be given to the biomolecule’s orientation, conformational structure, and arrangement upon the nanomaterial surface to ensure functionality. At the cell level, complexity increases further as cellular mechanisms and interactions may be considered to govern the bionanoconstruct recognition event. Forms of bionanoconstruct uptake and transport through diverse intracellular trafficking pathways must also be considered, and a complex decision-making process undertaken by the cell is expected in response to the recognition event. Though we do not explicitly outline them here, one can consider increasing levels of complexity (at the tissue, organ, and system levels). Finally, at the organism level, the outcome of a particular bionanoconstruct targeting experiment *in vivo* will be examined in terms of the biodistribution of these constructs in a given tissue or organ, and at this level, the role of biological barriers such as the blood brain barrier, as well as the potential for recognition of the construct as a foreign entity by the organism’s immune system, must be considered.

However, despite the significant progress in bioconjugation research, the exploitation of targeted bionanoconstructs *in vivo* has been limited.^21,22^ While the concept of active targeting may, in principle, be considered simple, in reality, programming the *in vivo* behavior of nanomaterials through the conjugation of biomolecules is exceptionally challenging and faces numerous levels of complexity (Figure 1). To go beyond trial and error-based efforts in the pursuit of active targeting and to achieve the desired clinical outcomes *in vivo*, approaches that bridge the gap between the molecular architecture of the nanomaterial surface and the biological identity of the construct are required. For some years, our understanding of bionanoscale recognition has not provided sufficient insight to meaningfully guide the rational design of bionanoconstructs; that is now, however, about to change.

As the complex cellular mechanisms governing biological recognition at the nanoscale are unraveled, it is becoming apparent just how intricate the issues underlying the biological recognition of bionanoconstructs are and how difficult it is to impart a favorable, functional identity to the construct.^23^ It is now understood that simply grafting a biomolecule, which is recognized in isolation by a target cell, to the nanomaterial surface does not lead to a productive biological identity, as the identity and activity of the bionanoconstruct are defined by a more collective interaction at the cell–nanomaterial interface.^24^ However, while much has been learned about what design parameters are undesirable and, thus, should be avoided, progress in understanding the requirements for bionanoconstruct recognition to be successful has been slow.^24−27^ In essence, access to key biological compartments and machineries is protected by a multitude of elaborate recognition mechanisms. To enter cells and access a productive endogenous pathway, it is insufficient for the bionanoconstruct to simply “stick” to the cell as a result of increasing the affinity of the nanomaterial for some molecular target particular to that cell. Gaining entry to the cell represents just the first hurdle; to execute some useful biological function, such as RNA delivery, the bionanoconstruct must escape the endolysosomal pathway by breaching barriers that have evolved over millions of years to prevent such access. Moreover, to attain passage across biological barriers such as the blood brain barrier or intestinal epithelium, the bionanoconstruct must pass even more elaborate recognition checks that involve multiple complex interactions.

Significantly, we now know that the biological recognition of surface architectures presented by bionanoconstructs in a physiological environment requires not just the avoidance of nonspecific adsorption but also the positive implementation of specific architectures which, by presenting appropriate collective interactions, act as the “key” to access highly regulated and protected biological gateways. Real progress is being made on the bionanoscale recognition front, and the cellular locks guarding biological gateways are now being dissected and understood; thus, this strategy will become a realistic agenda in the near future. As these realizations have materialized, it has also become evident just how (perhaps even innocently) ambitious early approaches were in developing bionanoconstructs for effective *in vivo* targeting. In this review, we discuss the properties of the surface architecture which are central to bionanoconstruct recognition and, thus, require rigorous control during preparation. We also emphasize the need for careful characterization of engineered bionanoconstructs and call attention to the challenges presented by population heterogeneity.

## 2 The Surface Molecular Architecture Matters

In biology, the cellular recognition of nanoscale objects, such as vesicles or viruses, is governed by the specific molecular architecture presented upon their surfaces. We believe that this also applies to bionanoconstructs: their precise surface molecular architecture defines their interactions with living systems, and changes in the surface architecture trigger different cellular responses.^23^ Controlling the biological activity of a bionanoconstruct is not possible without control over its surface molecular architecture. We, therefore, believe that a rigorous engineering strategy for the preparation of bionanoconstructs is necessary to ensure that the properties of the surface architecture central to recognition are controlled.

Of course, one must also pay consideration to the quality of the core nanomaterial upon which the architecture is engineered. All of the points we will outline in relation to the design and control of the surface molecular architecture would be rendered meaningless if applied to suboptimal nanomaterials presenting physicochemical defects. The core nanomaterial at the heart of the bionanoconstruct is by no means an inactive scaffold and should not be overlooked, as it too will influence the biological behavior of the construct and the overall therapeutic outcome.^28^ Driven by a very active community, research in the field of nanomaterial synthesis and characterization is progressing rapidly, and novel synthetic strategies which permit increased control over the size, shape, chemical composition, and polydispersity of nanomaterials are being developed. Similar consideration should also be given to the quality and integrity of the biomolecules to be used in the construction of the bionanoconstruct. The above illustrates just how dependent the success of targeted nanomedicines is on close, interdisciplinary collaboration.

### 2.1 Accessibility of Recognition Motifs

**Figure 2:**
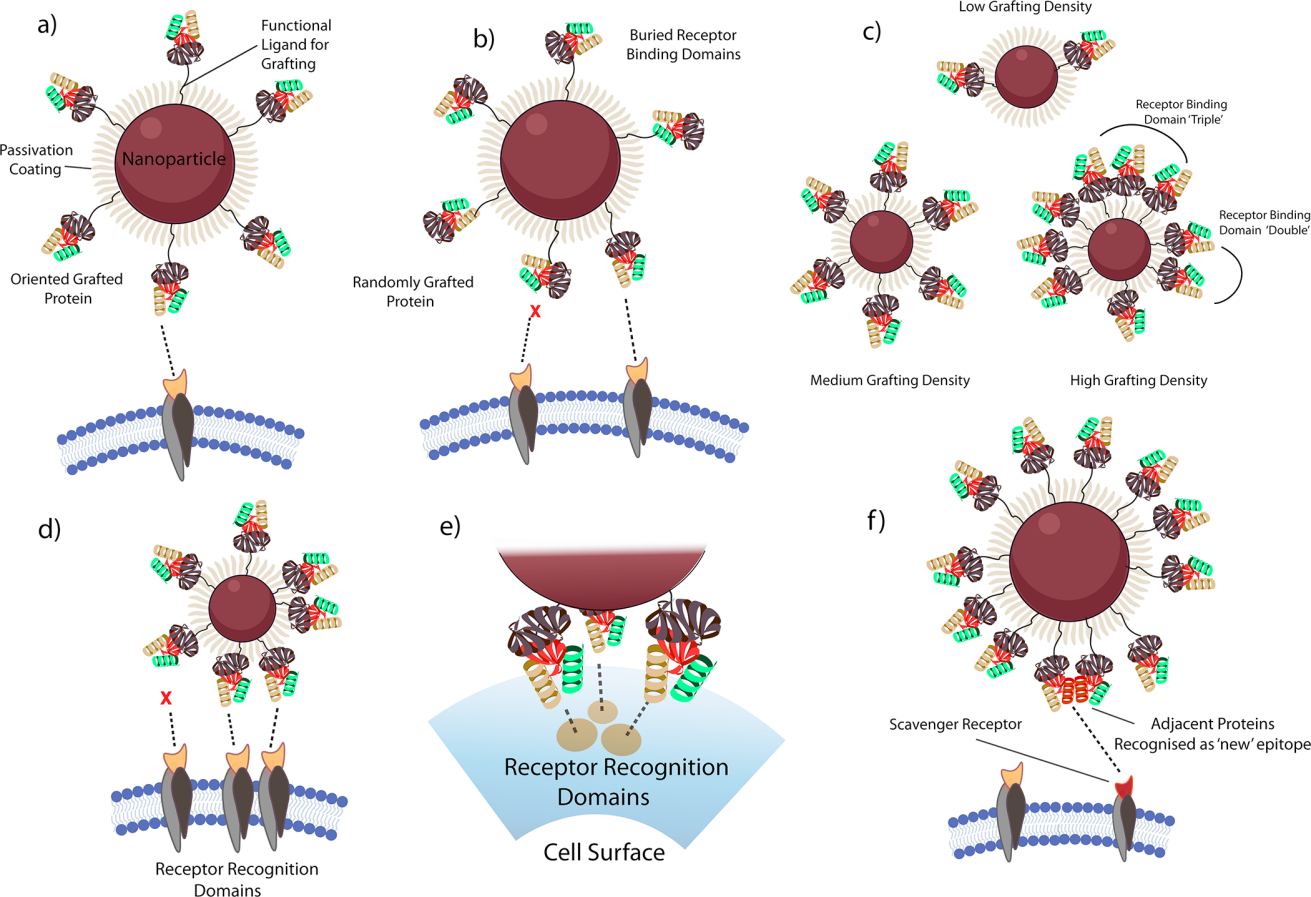
Factors to be considered in the design of bionanoconstruct surface architectures. (a) Nanoparticle with the oriented, grafted protein with a controlled intermediate surface density. The grafting is carried out such that the receptor-binding domains of the grafted protein are all oriented toward the exterior and are all available for binding to the target receptor while the diversity of unwanted exposed motifs is limited. Exposed regions of the nanoparticle are passivated against corona formation by an antifouling layer of, for example, polyethylene glycol. (b) As in part (a) but with protein grafted through some form of uncontrolled coupling chemistry which results in many of the grafted proteins presenting at the surface with an unsuitable orientation for target receptor binding. (c) Illustration of the effects of increasing protein graft density on epitope presentation. At higher graft densities, the potential for the presentation of groups of epitopes in clusters of doubles or triples increases significantly. (d and e) Illustration of the recognition by receptor doubles and triples. Immobilised proteins must be of sufficient proximity to one another to allow simultaneous interaction with receptors at the cell surface. (f) Potential implications of restricting the degrees of freedom through protein grafting. Free proteins in solution may undergo transient protein–protein interactions, but such interactions may be more long-lived on the surface of a bionanoconstruct, resulting in the possibility of “new” motifs being recognized by off-target receptors.

The most widely adopted strategy in the quest for the targeted delivery of nanomaterials is to conjugate an appropriate targeting ligand, complementary to a biomolecule expressed uniquely or preferentially by the cell type of interest, to the surface. Ligands interact with their targets in a highly specific manner through defined recognition motifs present within their molecular structure. Therefore, to prompt recognition by the cell type of interest, it is not sufficient for the bionanoconstruct to simply bear the targeting ligand; rather, it must display the active recognition motif. As an example, Figure 2a and b illustrates a nanoparticle–protein construct interacting with a target receptor on the cell surface. This relatively simple model demonstrates that the orientation of the conjugated ligand and accessibility of its recognition motif strongly impact the bionanoconstruct’s capacity to interact with its target receptor and, thus, its ability to execute its intended purpose.

Grafting of targeting ligands with a controlled, functional orientation (Figure 2a) produces a more defined and uniform surface architecture compared to the uncontrolled, random orientation (Figure 2b).^11,29,30^ This has been shown to improve the efficiency of the resulting bionanoconstruct in targeting applications *in vitro*.^31−34^ Due to the enhanced recognition capacity afforded, we strongly advocate for oriented grafting strategies. A wide range of strategies for regioselective grafting are available,^1,35−37^ with the simplest relying on the exploitation of naturally present reactive groups, such as thiols, within the targeting ligand structure. Other more complex strategies involve the incorporation of non-natural bio-orthogonal groups through cellular engineering^38^ or the enzymatic modification of proteins.^37,39−44^ Despite their ubiquity, we believe that random grafting approaches, such as those exploiting amine–carboxyl coupling via carbodiimide/sulfo-*N*-hydroxy succinimide chemistry, are not the future for nanomedicine, even if they remain convenient strategies for other applications where such stringent levels of control are not required. Such strategies can result in the targeting ligand conjugating to the nanomaterial surface in an inactive orientation, with access to its recognition motif blocked (Figure 2b). In fact, it has been shown that such recognition motif inaccessibility may be the most predominant result when employing random, uncontrolled conjugation strategies.^45^ In addition to a reduction in the presentation of the desired recognition motif, uncontrolled conjugation strategies may also lead to the undesirable exposure of other biologically active motifs, due to misorientation of the targeting ligands. For example, the conjugation of antibodies to nanomaterials via the antigen-binding fragments rather than the crystallizable fragment (Fc) domain results in presentation of the Fc domain at the surface. This can result in the bionanoconstruct undergoing off-target interactions with Fc receptors or proteins from the complement system, triggering unwanted biological responses.

Beyond assuring that the targeting ligand adopts the appropriate orientation, conjugation strategies should be designed to account for more subtle factors related to the accessibility of the recognition motif, such as its degree of freedom in relation to the nanomaterial surface. The degree of freedom of the conjugated ligand is largely governed by the molecular linker that connects the ligand to the nanomaterial surface. The length of this molecular linker becomes important if, for example, the target of interest is located in an environment where steric limitations preclude a close approach of the bionanoconstruct. In this instance, longer molecular linkers should be employed to conjugate the targeting ligand to the nanomaterial surface, to impart greater mobility to the ligand such that it may access and bind its target more readily.^45,46^

### 2.2 Mitigating Cryptic, Anomalous Epitopes

It is well-established that the activity of biomolecules is highly dependent on their conformational state. Therefore, when preparing bionanoconstructs, immobilization of the targeting ligand on the surface of the nanomaterial must not result in disruption of its structure if the desired activity is to be conferred.^30,47^ Preserving the structural integrity of targeting ligands upon conjugation is not only important in maintaining their intended function, but it also reduces the possibility of the grafted ligand displaying anomalous behaviors. Distortions of the targeting ligand structure can result in the exposure of hidden motifs, termed cryptic epitopes, which impart a different biological identity to the bionanoconstruct and may result in the bionanoconstruct engaging in off-target activity or eliciting unwanted immune or inflammatory system responses.^48−51^ Distorted targeting ligands may also prompt recognition and removal of the bionanoconstruct by scavenger receptors, a heterogeneous family of receptors capable of identifying a diverse range of both endogenous, damage-associated molecular patterns (DAMPs) and exogenous, pathogen-associated molecular patterns (PAMPs).^52^ Conjugation strategies must therefore be meticulously designed to mitigate damage to the targeting ligand structure. This involves careful consideration of details such as the preparation of the nanomaterial surface prior to conjugation. For example, when working with inorganic or hydrophobic nanomaterials, it may be preferable to passivate the surface with hydrophilic molecules prior to conjugation to prevent damaging adsorption of the targeting ligand to the bare nanomaterial surface.

### 2.3 Surface Density and Multivalency of Targeting Ligands

The surface density of conjugated targeting ligands is another key parameter of the surface molecular architecture that must be considered.^53^Figure 2c shows three different levels of grafting density at the nanomaterial surface, which we have classified as low, medium, and high. These are nonquantitative designations but can be understood as ranging from only a few sparsely conjugated targeting ligands to something approaching a close-packed monolayer.

At the most basic level, the more targeting ligands present on the nanomaterial surface, the greater the probability that the bionanoconstruct will engage with its intended target. Additionally, the presentation of an increased number of targeting ligands increases the probability of the bionanoconstruct engaging with multiple receptors at the cell surface simultaneously. This concept is illustrated in parts d and e of Figure 2, which showcase multivalent interactions of recognition motif “doubles” and “triples”, respectively. While likely to be distinguished as distinct entities from the endogenous free ligand by the cell, these multivalent architectures are known to enhance the apparent affinity of the bionanoconstruct for its target^54−57^ and, thus, may be necessary in order for the bionanoconstruct to compete effectively with the endogenous ligand. However, increasing the surface ligand density beyond a certain point can become counterproductive and begin to incite negative effects. First, if the bionanoconstruct demonstrates excessive affinity and interacts with its target too strongly, it is difficult to imagine that uptake, if it occurs, will follow the expected cellular pathway, as the bionanoconstruct is unlikely to dissociate from its binding partner in a comparable fashion to its endogenous counterpart. Demonstrating an excessively high affinity for the target of interest can also reduce the specificity of the bionanoconstruct, as it will interact with every cell presenting the target, even those exhibiting the target at low expression levels. Moreover, the multivalency will amplify the weak, nonspecific interaction between biomolecules, inducing nonspecific accumulation.

Conversely, it is also possible that the affinity of the bionanoconstruct for its target receptor could be compromised by excessively increasing ligand density, as reduced distances between adjacent targeting ligands may induce steric limitations that preclude access to the active recognition motif. This effect can be counterbalanced, in part, by adjusting the length of the molecular linker employed to conjugate the targeting ligands to the nanomaterial surface.^45^ The immobilization of targeting ligands in close proximity to one another on the nanomaterial surface may also result in the formation of novel recognition motifs that are identified and processed by the living organism in a manner different to that intended. These novel motifs may impart a distinct biological identity to the bionanoconstruct, prompting it to undergo off-target activities. It is also possible that such motifs may not be recognized nor tolerated “as self” by the living organism but identified as foreign molecular patterns by scavenger receptors and, thus, removed (Figure 2f).

While multivalent strategies represent the most commonly employed by the community, there have been studies in which intermediate ligand densities below nanomaterial surface-saturation levels were shown to be preferable in promoting target binding and cellular uptake.^58−60^ Certainly, when comparing the ligand densities of engineered bionanoconstructs to their natural viral counterparts, Alkilany et al. identified that typically, much higher ligand densities are deployed to accomplish the targeting of nanomedicines; an approach not adopted by viruses in order to optimize both infectivity and evasion of the host’s immune system.^61^ To this end, novel strategies which exert greater control over the ligand density of nanomaterials are emerging, permitting conjugation of a discrete number of ligands upon the surface and thus fine-tuning of the final construct’s biological behavior.^62^ Ultimately, when considering the surface ligand density, a balance must be struck between the affinity of the bionanoconstruct for its target and the construct’s overall viability. It is also likely that the optimal ligand density is specific to the particular targeting ligand, target receptor, and application in question and will need to be established on a trial and error-based approach until a deeper understanding of the mechanisms governing biological recognition at the nanoscale is obtained.

Of course, constructing a functional architecture on the surface of the nanomaterial will be accompanied by an increase in size and an alteration in shape of the final construct. While some general trends have emerged surrounding the ideal nanomaterial size and shape for therapeutic application, it is likely that the optimal parameters will be specific to the particular biological target and desired clinical outcome.^63−66^ Moreover, it has been observed that a broad range of nanomaterial sizes accumulate in the liver and spleen,^21^ with the exception of, to some extent, ultrasmall nanoparticles displaying no hard corona.^67−69^ Thus, we believe that the key to controlling the biodistribution of bionanoconstructs lies in the control of their biological interactions through customized surface molecular architecture, rather than through control of the size of the final object.

## 3 The Surface Molecular Architecture Is Influenced by the Surrounding Environment

In addition to being nontoxic and biocompatible, an ideal bionanoconstruct should leave no footprint on a living system except for that related to the conjugated targeting ligand. In this regard, the bionanoconstruct must resist alteration by the surrounding environment. Physiological systems, in particular, are highly complex and dynamic in nature and can exert significant influence over the bionanoconstruct’s functional surface architecture and overall stability. Measures must, therefore, be taken to attenuate the influence of the surrounding environment and ensure the preservation of the bionanoconstruct’s intended activity and biocompatibility.

### 3.1 Biomolecular Corona Formation

**Figure 3:**
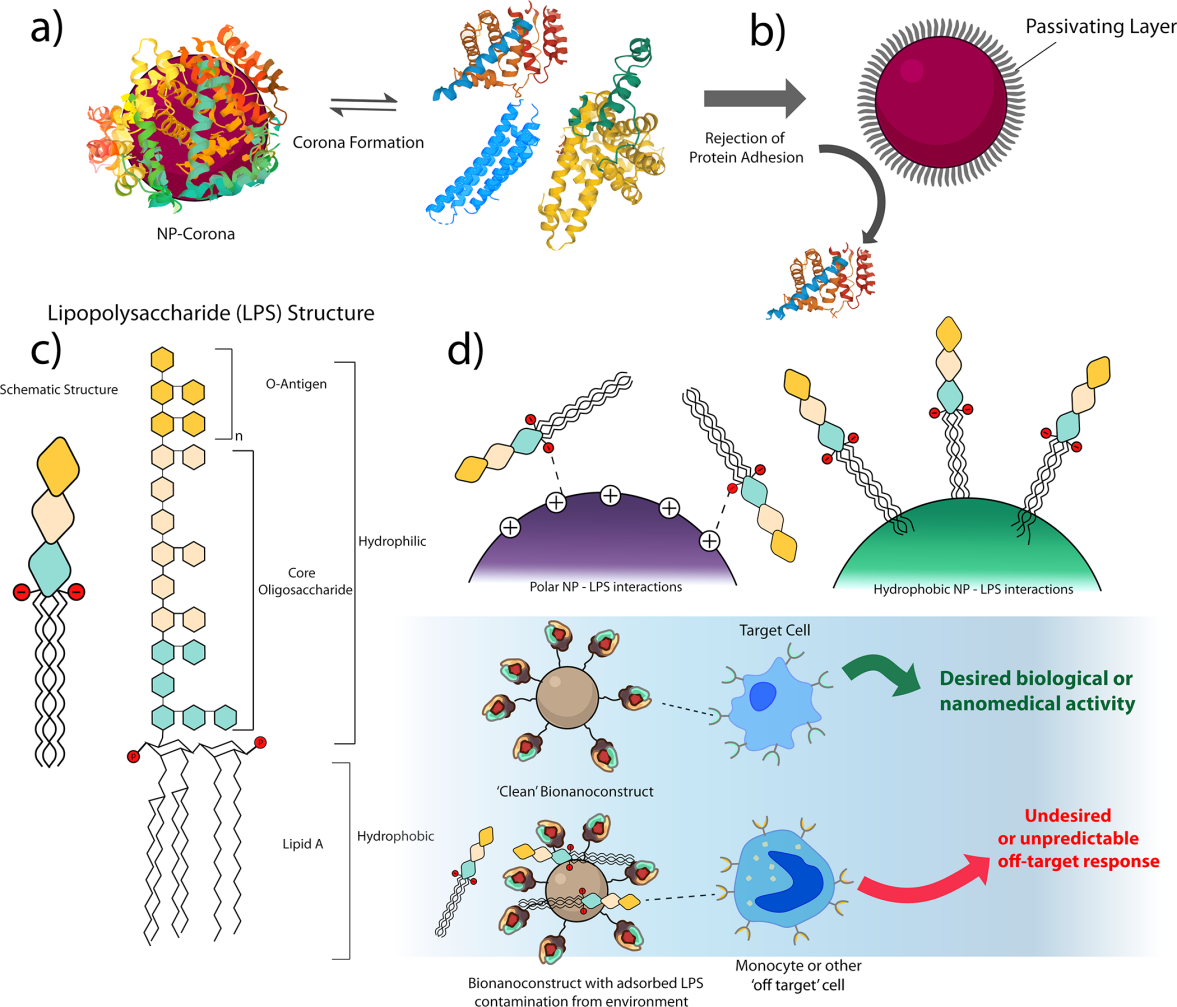
Influence of the surrounding environment on the surface molecular architecture. (a) NP–corona formation on a bare NP and (b) the “stealth” effect of surface passivation with, for example, polyethylene glycol (PEG) groups. Passivation reduces nonspecific adsorption of proteins and, hence, limits corona formation in biological milieu. (c) Cartoon structure of lipopolysaccharide (LPS), showing the O-antigen, oligosaccharide, and lipid A components. (d) Top: LPS may adhere to the surface of polar NPs through electrostatic interactions or via hydrophobic interactions through the lipid A component. Bottom: the presence of LPS in otherwise functional bionanoconstructs can lead to undesired inflammatory responses *in vivo*. Such responses can range from mild to highly severe in nature and may be entirely unrelated to the activity of the bionanoconstruct under consideration. They may also cause the intrinsic inflammatory response of a bionanoconstruct to be unmeasurable.

It is well-established that once nanomaterials are dispersed in a biological fluid, their surfaces will be modified through the spontaneous adsorption of surrounding biomolecules, forming a biomolecular corona.^70^ It is widely accepted that this corona ultimately determines the biological identity of the nanomaterial and controls its fate *in vivo*.^24^ If allowed to form at the surface of bionanoconstructs, the biomolecular corona can eliminate the desired function of the construct by masking the targeting ligands central to their activity (Figure 3a).^24,71−73^ Moreover, the adsorbed biomolecules impart new recognition motifs to the bionanoconstruct, which may prompt uptake by off-target cells^26,74−78^ or trigger a diversity of unintended biological mechanisms.^79−82^ If left unchecked, these newly acquired motifs imparted by the biomolecular corona effectively reprogram the biological identity of the bionanoconstruct, resulting in a complete loss of control of its activity.

The biomolecular corona has been intensively studied; however, awareness of its significance in determining the biological identity of nanomaterials has only emerged within the past decade, and its precise nature and impact *in vivo* remain elusive. To add to the complexity of the issue, the biomolecular corona is a dynamic layer^70,83^ whose composition is strongly influenced by the particular surrounding environment. Notably, it has been demonstrated recently that the biomolecular corona formed *in vivo* may be different to that formed *in vitro*, both in the identity and number of biomolecules adsorbed, due to the influence of a dynamic flow environment.^84−87^ This presents a significant barrier to the translation of *in vitro* results to a practical *in vivo* setting.

To circumvent the difficulty in anticipating the biological activity of bionanoconstructs following biomolecular corona formation, ideally, the bionanoconstruct should be designed such that adsorption of this additional layer is obstructed. While no strategy currently exists to fully preclude biomolecule adsorption to nanomaterials in a physiological environment, several approaches have been developed to minimize the phenomenon.^68,76,88,89^ One of the most common strategies involves coating the nanomaterial surface with a layer of hydrophilic polymers such as poly(ethylene glycol) (PEG). This layer passivates the nanomaterial surface and reduces nonspecific adsorption of biomolecules by acting as a steric shield.^88−93^ This hydrophilic layer, illustrated in an idealized format in Figure 3b, prevents strong interactions from occurring between circulating biomolecules and the nanomaterial surface, and it allows formation of only a transient soft corona. This soft corona, comprised of weakly interacting biomolecules that exchange rapidly with the nanomaterial surface, does not impart a prevalent biological identity to the bionanoconstruct, unlike the static hard corona formed at the surface of unpassivated nanomaterials.^77,94^ To reach a satisfactory level of passivation, particular attention must be paid to the quality of PEGylation. It has been reported that in order to be effective, the surface PEG density must surpass a particular threshold, which depends on factors such as the surface curvature of the nanomaterial and the polymer chain length.^95−97^ Since it can be difficult to obtain a sufficiently high passivation density with long polymers due to steric limitations, shorter, less bulky ligands are often used as backfillers to occupy spaces inaccessible to the long polymers, thus reinforcing the coating.^46,76,77,94^ In addition to PEGylation, a number of other strategies have been developed to passivate the surface of nanomaterials including coating with zwitterionic molecules,^68,98^ saccharides,^99^ and other biopolymers such as polyoxazolines, polysarcosines, polymethacrylamides, and polyglycerols.^100,101^

### 3.2 Bionanoconstruct Aggregation and Contamination

While the implication of the biomolecular corona is intensively discussed in the literature, there are a number of other confounding factors that can disrupt the biological identity and compatibility of bionanoconstructs that are often neglected. Consideration should be given, for example, to the colloidal state and stability of the bionanoconstructs in physiological media. Beyond the danger of vessel blockage posed by circulating aggregates, it is known that the size of nanomaterials strongly influences their biodistribution *in vivo*.^102,103^ It is, therefore, important to assess the integrity of the bionanoconstruct dispersion in conditions that mimic the physiological environment. In this respect, it should be recognized that upon administration *in vivo*, the bionanoconstruct will encounter a variety of conditions and barriers, all of which may influence the state of the colloidal dispersion.

Another confounding factor that warrants attention concerns microbial contamination of the bionanoconstruct formulation during preparation. Microbial contamination of the surface can lead to misinterpretation of the bionanoconstruct’s compatibility, as a poor safety profile is falsely accredited to the construct rather than the contaminant. Particular caution is required in the case of lipopolysaccharide (LPS, detailed in Figure 3c), a well-characterized surface antigen of Gram-negative bacteria that initiates a potent immune response *in vivo*.^104^ LPS is a ubiquitous environmental contaminant that may persist even in the absence of live bacteria.^105^ Owing to its pro-inflammatory properties, the US Food and Drug Administration prescribes a limit of <0.5 Endotoxin Units (EU) of LPS per milliliter in pharmaceuticals, food products, and medical device extracts. There is considerable evidence, however, that a much lower limit should be pursued in bionanoscience, as immobilization of LPS on the surface of nanomaterials results in a high local concentration of the antigen and, thus, amplification of its recognition and impact (Figure 3d).^106^ Due to its amphiphilic nature, LPS adsorption to both hydrophobic nanomaterials (via the hydrophobic lipid A component) and hydrophilic nanomaterials (via phosphate moieties) is readily facilitated through a variety of Coulombic and van der Waals interactions (Figure 3d). These interactions may be suppressed to varying degrees by controlling the conditions of the suspension medium, such as pH and ionic strength. The high thermostability of LPS renders the molecule resistant to conventional sterilization techniques, and only prolonged heating at temperatures above 180 °C is effective in its removal.^106,107^ Since the application of such methods would dismantle the bionanoconstruct’s physicochemical properties and stability, precautions must be taken to mitigate LPS contamination during its preparation. This requires chemists to adopt rigorous aseptic techniques in their synthetic procedures, such as utilizing laminar flow hoods, assessing all reagents for contamination prior to use, and conducting appropriate sterilization of all glassware and equipment.

## 4 The Surface Molecular Architecture Must Be Characterized

It is well-established that a lack of careful characterization represents a significant barrier to the translation of nanomaterial-based therapeutics from bench to bedside.^108−113^ Given all of the complications in generating effective bionanoconstructs, including the engineering of a functional surface architecture and precluding derivatization of this architecture in biological milieu, comprehensive characterization of the bionanoconstruct on several fronts is imperative to achieve the desired clinical outcome. On one side, methodologies must be developed to characterize the surface molecular architecture of bionanoconstructs, to obtain qualitative and quantitative information on the composition and organization of this functional framework *in situ*. It is also critical to identify the key molecules and cellular pathways that are involved in bionanoconstruct recognition and, thus, regulation of the construct’s biological activity. Without characterization along both of these fronts, the underlying mechanisms governing bionanoconstruct performance cannot be comprehended. Understanding the construct itself also informs the suitability of the synthetic strategy employed and permits correlation of the bionanoconstruct’s behavior to the anatomy of its surface. This, in turn, allows for informed evaluation, rational modulation, and reproducibility of the bionanoconstruct’s performance.

### 4.1 Characterization of the Surface Molecular Architecture Composition

Considering that recognition and the resulting biological performance will be governed by the molecular architecture presented upon the nanomaterial surface, bionanoconstructs should not be deployed in ignorance of the precise composition of this framework. Each of the surface attributes we have highlighted as pertinent to the biological identity of the bionanoconstruct warrants careful characterization and evaluation. Since no solitary analytical technique can provide complete characterization of the bionanoconstruct, a combination of methods must be used to unveil all of its properties. It should also be recognized that the identity of the core nanomaterial and conjugated targeting ligand will dictate which characterization techniques may or may not be applied when evaluating the construct and the level of complexity encountered in their study.

First and foremost, conjugation of the desired targeting ligand to the surface of the nanomaterial should be verified, for example, through chromatographic, electrophoretic, or spectroscopic means.^114−123^ Such experiments can also provide insight into the average nanomaterial–targeting ligand ratio of the bionanoconstruct. When performing such assessments, it is essential that the bionanoconstruct is thoroughly washed to avoid interference from any free ligand in suspension and, thus, misinterpretation of results. Indeed, beyond simply verifying conjugation of the desired targeting ligand, parameters central to its intended function must be characterized, namely the structural conformation of the ligand and its precise orientation on the nanomaterial surface, as discussed previously. A variety of techniques may be used to investigate the structural conformation and integrity of the targeting ligand upon conjugation to the nanomaterial, such as circular dichroism, UV–visible absorption spectroscopy, fluorescence spectroscopy, Fourier-transform infrared spectroscopy (FTIR), and nuclear magnetic resonance spectroscopy (NMR).^47,119,124−137^ These methods may also be applied to monitor alterations in the conjugated ligand’s structure in response to variable physicochemical properties of the surrounding environment, such as pH or ionic strength. Techniques capable of revealing the precise orientation of the targeting ligand on the nanomaterial surface are more limited, but they include NMR,^121,134,135,138,139^ fluorescence resonance energy transfer (FRET) studies,^140^ and proteolytic-mass spectrometry analyses.^141−143^ The evaluation of each of the surface architecture parameters informs the suitability of the synthetic strategy employed and whether amelioration of the strategy may be required. Affirming targeting ligand presence, conformation and orientation upon the nanomaterial surface can also point to the likelihood of the bionanoconstruct demonstrating the desired activity and permits identification of suitable candidates for further structure–activity relationship studies.

Beyond the need for careful characterization of the composition of the bionanoconstruct’s functional surface architecture, there are several other features of the composite structure that should be investigated at various stages throughout the engineering process. Such features include, for example, the density of ligands used in the passivation of the nanomaterial surface and their ability to preclude corona formation and the overall physicochemical properties, such as the hydrodynamic diameter, mass, shape, surface area, zeta potential, colloidal stability, and purity of the prepared bionanoconstruct. The incorporation of methodologies capable of probing these features to the bionanoconstruct development workflow is imperative, as they too influence the pharmacokinetic profile and behavior of the bionanoconstruct during application. For a comprehensive discussion of the characterization of nanomaterials and their bioconjugates, the reader is referred to reviews prepared by Sapsford et al.^29^ and Khorasani et al.^111^

### 4.2 Characterization of the Surface Molecular Architecture’s Biological Activity

**Figure 4:**
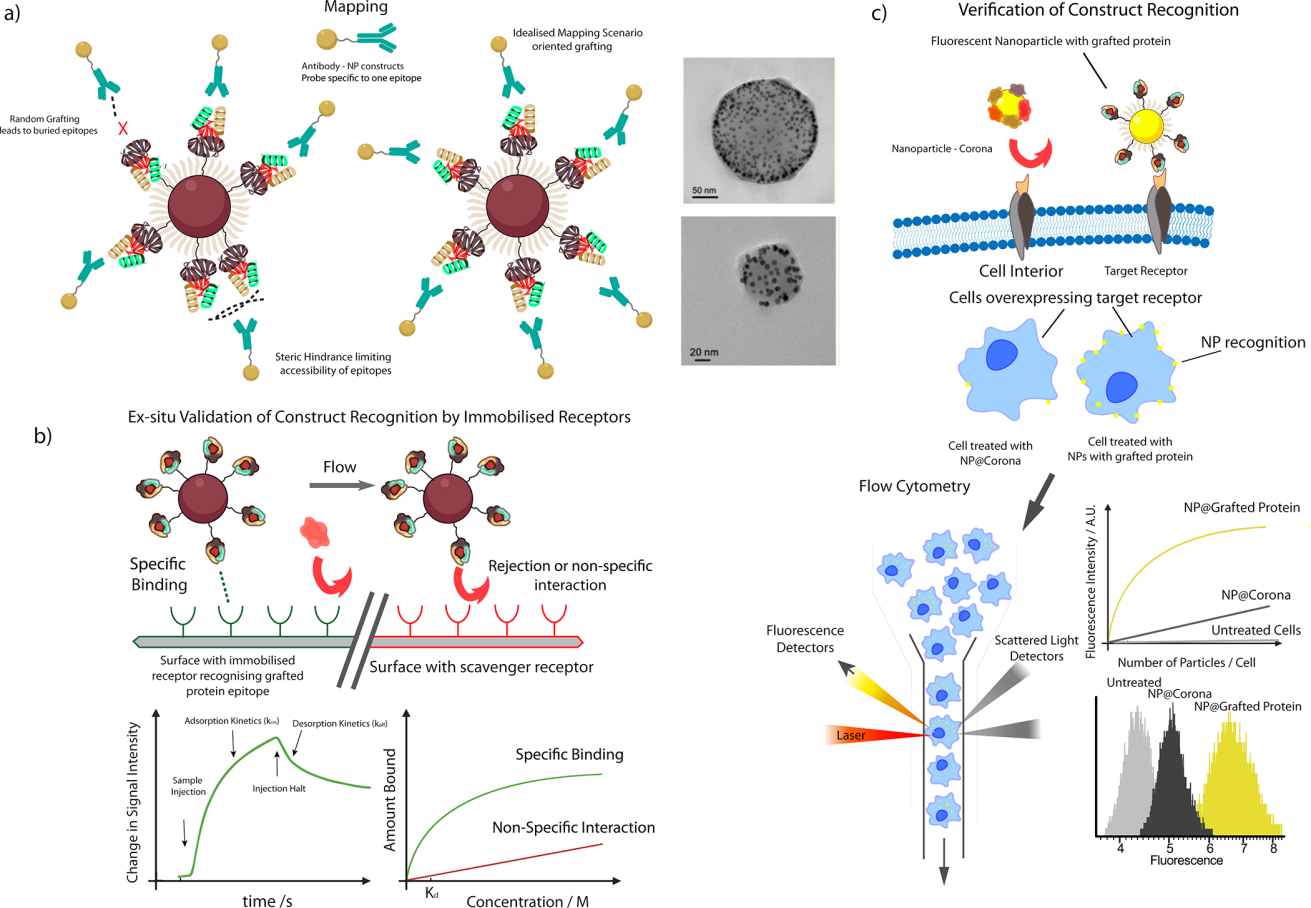
Characterization workflow for bionanoconstructs. (a) Mapping of epitopes illustrated by the antibody–NP mapping constructs. Left: challenges associated with mapping are largely associated with steric hindrance impeding the mapping constructs from binding to their target epitopes. Right: In the idealized scenario, all exposed epitopes of the grafted protein are available for binding to mapping constructs and can be quantified by TEM imaging (Reprinted with permission from ref (45). Copyright 2017 American Chemical Society). (b) Illustration of *ex situ* receptor recognition testing of bionanoconstructs using immobilized receptor layers in techniques such as quartz crystal microbalance measurements and surface plasmon resonance studies. These techniques allow for confirmation that a bionanoconstruct displays “on-target” binding as well as minimizing or eliminating off-target interactions with selected receptors, such as scavenger receptors. Bottom left: a typical adsorption–desorption curve associated with specific binding of a construct to an immobilized receptor, which allows for the binding kinetic to be evaluated. Bottom right: Observed isotherm behavior for specific binding vs nonspecific interaction of bionanoconstructs with receptors. (c) Investigation of bionanoconstruct interaction with cells *in vitro* through receptor-binding experiments on cells engineered to overexpress a particular receptor which recognizes the bionanoconstruct or directly on targeted cells. The measured interaction levels can be referenced to controls of nonspecifically engineered particles with adsorbed biomolecular coronas. As discussed in the text, the interaction level may be confirmed through various microscopy techniques; here we illustrate the use of flow cytometry to quantitatively analyze uptake in a cell-by-cell fashion using fluorescent NPs.

Toward identifying bionanoconstructs with favorable surface architecture composition and, thus, those candidates that may demonstrate the desired biological activity, our group advocates the use of an antibody-based labeling approach to map out the surface architecture of individual particles (Figure 4a). This epitope mapping strategy involves the engineering of immunonanoprobes, comprised of an antibody that binds to a specific site of a particular protein of interest, conjugated to some nanoscale reporter that permits identification, traditionally gold nanoparticles or quantum dots. Our group has demonstrated the utility of this immunolabeling strategy both in the study of the biomolecular corona and in the characterization of engineered bionanoconstructs.^45,74,144,145^ The technique holds the potential to characterize several features of the bionanoconstruct surface architecture concurrently, confirming conjugation of the desired targeting ligand to the nanomaterial surface, verifying that the ligand is oriented correctly with the key recognition motif outwardly presented and permitting quantification of the recognition motifs available. The technique also has the ability to discern the spatial arrangement and distribution of targeting ligands upon the nanomaterial surface. The precise distribution of targeting ligands on the bionanoconstruct surface is an important parameter to consider, as the particular arrangement will modulate biological activity and therapeutic output by exerting influence over ligand flexibility, recognition motif accessibility, and target affinity.

Further characterization beyond unveiling the composition of the surface architecture is required, however, to truly understand how the bionanoconstruct behaves within a physiological system. Simply affirming compositional properties such as targeting ligand conjugation, structural conformation, and orientation merely acts as a proxy to predict the potential biological activity of the bionanoconstruct; it cannot conclusively ensure it. Toward understanding how the bionanoconstruct might behave in an *in vivo* setting, prerequisite *in vitro* studies dedicated to exploring the relationship between the surface architecture of the bionanoconstruct and its biological activity must be conducted. Ultimately, the diagnostic or therapeutic efficiency of an engineered bionanoconstruct will depend on its ability to interact with its intended target. Dedicated interaction studies are therefore required to ascertain whether the targeting ligand conjugated to the nanomaterial surface is successfully recognized and bound by its target and, thus, whether the bionanoconstruct is likely to demonstrate the desired activity. Quartz crystal microbalance with dissipation monitoring (QCM-D) and surface plasmon resonance spectroscopy (SPR) are examples of two powerful surface sensing techniques that have the capacity to study such biomolecular interactions (Figure 4b).^70,146−154^ Of course, the diagnostic or therapeutic activity of many bionanoconstructs will also depend on their successful cellular uptake and correct intracellular distribution upon interaction with the target. This is particularly true in cases where the bionanoconstruct has been designed to act as a carrier of molecules of interest, such as drugs, nucleic acids, or contrast agents. The cellular uptake and intracellular distribution of nanomaterials and their bioconjugates are commonly assessed by techniques such as flow cytometry (Figure 4c), confocal laser scanning microscopy, transmission electron microscopy, Raman spectroscopy, and inductively coupled mass spectrometry.^155−174^ When performing any receptor interaction, cellular uptake, or intracellular distribution study, consideration must be given as to whether the microenvironment of the target is adequately represented, to ensure correct interpretation of the bionanoconstruct’s activity. The conditions of the microenvironment surrounding the target will influence the physicochemical properties of the bionanoconstruct, which in turn determine whether the construct is recognized by its target and internalized by the cell, and by which intracellular route the construct is trafficked.^175^

In addition to assessing the interaction and uptake of the bionanoconstruct with its intended target, it should be investigated whether the construct engages in any nonspecific, off-target behaviors. It can be particularly useful to evaluate the recognition of the bionanoconstruct by components of the immune system. For example, uptake of the bionanoconstruct by cells of the mononuclear phagocyte system may be assessed,^157−159,166^ and the recognition of the bionanoconstruct by scavenger receptors may be evaluated by biomolecular interaction techniques such as QCM-D or SPR (Figure 4b). Sequestration of the bionanoconstruct by such entities is not desirable, as it indicates that the bionanoconstruct is not tolerated by the physiological system and, thus, is an unsuitable candidate for practical, medical use. Upon demonstrating the desired activity, compatibility, and a lack of toxicity *in vitro*, the bionanoconstruct may be taken forward for *in vivo* assessment to establish the efficacy, safety, and pharmacokinetic profile of the construct using animal models. It is important to keep in mind that while successful results may be obtained in initial *in vitro* studies, and perhaps in prerequisite *in vivo* animal models, the translation of bionanoconstructs to a clinical setting is not guaranteed and remains a significant challenge.

### 4.3 Considering Bionanoconstruct Heterogeneity

An issue encountered with many conventional characterization strategies is that as the analysis is performed on the bulk formulation rather than on individual particles, they provide only a generalized interpretation of the bionanoconstruct surface composition, characterizing surface attributes with averaged values. This “one size fits all” approach is wholly inappropriate, as it hides the true nature of the bionanoconstruct formulation. Bionanoconstructs will exist as a distribution of distinct subpopulations, stratified on the basis of heterogeneities in the surface architecture of individual particles. Current conjugation strategies yield, at best, distinct subpopulations of bionanoconstructs demonstrating variations in the discrete number and distribution of targeting ligands conjugated to the nanomaterial surface. The probable state of bionanoconstruct surface composition estimated from currently available characterization techniques, therefore, does not reflect the true nature of the collective formulation, as it is derived from a diverse and complex mixture of states. Indeed, by definition, the average is not representative of extreme states that differ significantly from the generalized state.^176^ The concept of bionanoconstruct heterogeneity in terms of variable ligand stoichiometry is well-documented throughout the literature.^113,115,177−179^ The attachment of ligands to the surface of nanomaterials tends to follow a Poisson distribution, with unfunctionalized, monofunctionalized, and polyfunctionalized construct populations being produced. The extent of heterogeneity encountered within the bionanoconstruct formulation will be influenced by the strategy implemented in its preparation, and it should be recognized that beyond variable ligand density, heterogeneities can also exist in the conjugated ligand distribution, orientation, and recognition motifs presented, not to mention in the core nanomaterial dispersion itself.

Considering the biological system’s innate ability to discriminate small structural details at the molecular level, the existence of heterogeneities in the surface architecture of individual bionanoconstructs cannot be ignored.^108^ Heterogeneity within and across bionanoconstruct formulations will result in inconsistent, unpredictable, and irreproducible performance as individual subpopulations with unique surface compositions may elicit a distinct biological response.^180^ This presents difficulties in ascertaining the efficacy and safety profiles of the bionanoconstruct, as variations in surface properties central to performance reduce the proportion of constructs demonstrating the desired activity within the formulation. The existence of subpopulations exhibiting suboptimal or ineffective surface architectures may also trigger unexpected and potentially harmful immune system response or off-target reactivity, thus presenting an effective barrier to clinical translation. Therefore, there exists an urgent need to develop methodologies capable of characterizing surface architecture at the single particle level, toward identifying individual subpopulations and demystifying their biological significance. The epitope mapping strategy previously described represents a promising avenue on this front.

## 5 Conclusion

The last 30 years of research into the preparation of bionanoconstructs for nanomedicine has produced a vibrant and diverse interdisciplinary field, incorporating elements of nanomaterial synthesis, surface derivatization, biochemistry, and molecular biology. However, during this time, it has also been realized that the preparation of functional bionanoconstructs for effective *in vivo* targeting is not so straightforward as to simply conjugate an appropriate biomolecule to the nanomaterial surface. It is our belief that physiological environments, cells in particular, are extremely sensitive to minute variations in the surface architecture of bionanoconstructs, and precise control of this surface architecture is imperative in producing functional bionanoconstructs. To this end, surface biofunctionalization strategies must be designed to integrate our evolving understanding of bionanointeractions, and the suitability of these strategies must be validated with complete and careful characterization of the bionanoconstruct. Characterization of both the composition and activity of the bionanoconstruct is important, not only to validate the synthetic strategy employed in its preparation but also to relate the properties of the surface architecture to the biological behavior observed and to identify dominating parameters. The heterogeneity found within and across bionanoconstruct formulations is also something we believe important to characterize, as it can be misleading to correlate observed biological behavior with an averaged interpretation of the bionanoconstruct’s composition. The complexity of additional steps required in the control and characterization of the surface architecture we have described should not be regarded as a synthetic bottleneck but, instead, be seen as the way forward to achieve improved targeting efficiency of bionanoconstructs.

The concept that an ideal bionanoconstruct for active targeting should consist of a nanomaterial that is conjugated to a suitable biomolecule and presents a neutral footprint to the physiological environment remains the most common blueprint followed by the community. However, it is yet to be seen whether such a system can be effective in practice. Certainly, biological interfaces are multifunctional systems with their biological identity and activity defined by the synergy of all of their constituent parts. This suggests an alternative way of thinking, whereby instead of using isolated biomolecules to confer targeting capability, endogenous cell recognition motifs are mimicked to create a complete biological interface at the bionanoconstruct surface. The realization of such an approach will require a profound understanding of bionanointeractions, as well as perfect control of the surface molecular architecture in the engineering of bionanoconstructs. As an intermediate response, biomimetic strategies have emerged.^181−185^ While, in this case, the precise mechanisms of recognition are still unknown, at least partially, the functionalization of conventional nanomaterials with biologically sourced building blocks, such as cell membranes, vesicles, or viral capsids, provides the proper codes for nanomaterials to engage with the biological environment.
